# Health risk assessment of exposure to various vapors and fumes in a factory of automobile manufacturing

**DOI:** 10.1016/j.heliyon.2023.e18583

**Published:** 2023-07-22

**Authors:** Amir Hossein Khoshakhlagh, Saeid Yazdanirad, Hamid Reza Saberi, Pao-Chi Liao

**Affiliations:** a Department of Occupational Health, School of Health, Kashan University of Medical Sciences, Kashan, Iran; b Social Determinants of Health Research Center, Shahrekord University of Medical Sciences, Shahrekord, Iran; c School of Health, Shahrekord University of Medical Sciences, Shahrekord, Iran; d Occupational Health & Safety Department, Kashan University of Medical Sciences, Kashan, Iran; e Department of Environmental and Occupational Health, College of Medicine, National Cheng Kung University, 138 Sheng-Li Road, Tainan, 704, Taiwan

**Keywords:** Risk assessment, Cancer, Non-cancer, Vapors, Fumes automobile manufacturing

## Abstract

This study aimed to comprehensively evaluate the health risk of exposure to various vapors and fumes in a factory of automobile manufacturing. This study was performed in 2021 on 115 workers. Vapors and fumes were gathered by the adsorbent tubes of activated charcoal and mixed cellulose esters (MCE) membrane filter, respectively. The flow rate for vapors and fumes were between 0.05 and 0.20 L per min and 1 to 4 L per min, respectively. After preparing, samples were analyzed. To assess the non-cancer and cancer risk of the pollutants, the method proposed environmental protection agency (EPA) was applied. The total concentration of copper (1.031 ppm), manganese (0.114), and 2-butoxyethanol (91.767 ppm) were found to be higher than The threshold limit values (TLVs). The values of non-cancer risk (HQ) due to exposure to vapors of benzene (6.583), toluene (1.396), ethyl benzene (1.212), xylene (31.148), 2-butoxyethanol (89.302), 2-propanol (4.695), 1,2,3-trimethylbenzene (1.923), copper (2.336), manganese (715.82), aluminum (3.772), and chromium (107.066) were higher than the acceptable limit. Moreover, the estimated LCR for benzene (2.15 × 10^−4^), ethyl benzene (3.97 × 10^−4^), vinyl chloride (1.25 × 10^−4^), and chromium (2.11 × 10^−2^) were higher than the threshold risk level set by EPA. It is emphasized that preventive measures are performed.

## 1 Introduction

There are different harmful agents in the various work environment, which can affect the worker’s health [1,2]. The automobile producing companies are one of these workplaces. In these companies, there is a variety of occupational accidents and negative consequences affecting the health of the personnel [3]. Exposure to toxic air pollutants is one of the most ubiquitous hazards in this industry. Various processes, such as welding, washing, painting, and assembling, are performed in automobile-producing companies. In these processes, a variety of materials, such as metals, paints, solvents, adhesives, plastics, and polymers, are used. During the use of these materials, the workers are exposed to vapors such as volatile organic compounds, resin monomers, and particles [3,4].

Two important groups of workplace pollutants in automobile-producing companies include vapors and fumes. The exposure to vapors of volatile organic compounds in automobile-producing companies has different effects depending on the type of compound. These compounds can cause eye, nose, and throat irritation, breath shortness, headache, fatigue, nausea, dizziness, and skin problems in humans. Prolonged exposure to higher concentrations can lead to lung irritation as well as damage to the liver, kidneys, and central nervous system [5]. In addition, some volatile organic compounds such as benzene and ethyl benzene can cause cancer in humans. The carcinogenic effects of benzene exposure have been conclusively confirmed by the International Agency for Research on Cancer (IARC) [6]. Ethylbenzene is also suspected of carcinogenicity [6]. Moreover, the vapors of monomers, such as vinyl chloride, can be dangerous. There is vinyl chloride in some parts of automobile-producing companies. Exposure to this material is most strongly associated with non-malignant health effects, including in the liver, brain, and other organs [7]. Also, it has been recognized as a cause of liver cancer [7]. In addition to stated substances, the workers of automobile manufacturing are exposed to metal fumes due to welding and hot process. Some of these fumes include iron oxide, copper, manganese, zinc oxide, aluminum, and chromium. The effects of the fumes depend on the type of substance. Previous studies have reported that metal fumes can cause lung function impairment, obstructive and restrictive lung disease, cough, dyspnea, rhinitis, asthma, pneumonitis, and pneumoconiosis [8]. Some metal fumes, such as chromium, nickel, and manganese may pose more of a potential hazard than others [9]. The IARC classified some welding fumes, such as chromium, as possibly carcinogenic to humans [10].

To prevent the harmful effects of the stated materials, a health risk assessment must first be performed. So that the control measures are focused on exposures with high risk. The health risk assessment can increase awareness of human health in various workplaces [11,12]. The national research council (NRC) defined risk assessment as the determination of the potentially harmful health effects in humans because of environmental exposure to hazards [13,14]. Hence, risk assessment, as an essential tool for the prediction of the harmful health effects due to exposure to chemical compounds, contain four steps, including hazard identification, dose-response assessment, exposure assessment, and risk determination [15,16]. This method combines the exposure and dose-response data on chemical substances for computing the score of risk and determining the probability of adverse health effects [17,18].

The health risk assessment method has been used in previous studies to evaluate the health risk of various materials in the industry of automobile manufacturing. For example, Perez et al. investigated the health risk of diundecyl phthalate (DUP) as a high molecular weight phthalate plasticizer present in automobile interiors and concluded there was no expectation of a hazard to humans for any reasonable exposure scenario [19]. Nduka et al. assessed the health risk of cadmium, chromium, and nickel in car paint used at auto-panel workshops. The results of this study showed that exposures to these substances through inhalation, ingestion, and dermal contact may be of significant public health importance and can add to the body's burden of some carcinogenic [20]. Tong et al. evaluated the quantitative health risk assessment of inhalation exposure to automobile foundry dust. They concluded that the dust had various health risks among foundry workers in different types of workplaces [4]. Badjagbo et al. studied the health risks associated with benzene, toluene, ethylbenzene, and xylene (BTEX) compound exposures among automobile mechanics and painters. The results of this study indicated that the levels of the BTEX measured at all the garages were less than the established limits for occupational but benzene levels pose a potential cancer risk for the workers [21]. Tong et al. performed a study on the health risk assessment of ambient volatile organic compounds in automobile manufacturing processes. They concluded that there is a definite carcinogenic risk for exposure to benzene and ethylbenzene. Total non-carcinogenic risk in the paint shop also was the highest [3].

However, the health risk of some pollutants in the industry of automobile manufacturers has been not assessed in previous studies. Also, any comprehensive study has been not conducted on the health risk assessment of the main pollutants in this industry that simultaneously compare exposure to several pollutants, including vapors and fumes. Therefore, the present study aimed for the first time to evaluate the health risk of exposure to various vapors and fumes in a factory of automobile manufacturing.

## 2 Materials and methods

This study was conducted in 2021 on 115 workers of a factory of automobile manufacturers in the center of Iran for evaluating the health risk due to occupational exposure to vapors and fumes among them. Firstly, the researchers surveyed the production process and raw materials and consulted experts in this plant. Then, the places with the probability of exposure to vapors and fumes were determined and the subjects occupied in these places were identified. The aims and steps of the present study were explained to the persons and they were asked to participate in the research. All of the selected workers signed a consent form to participate in this study.

### 2.1 Site description

A factory of automobile manufacturers in the center of Iran was selected for this study. There are some parts in this factory, including research, development, production, and automobile sale. The production part comprises several shops, including a press shop, body shop, paint shop, assembly shop, pre-delivery inspection shop, and material storage shop. In this factory, various materials, such as metals, paints, solvents, adhesives, plastics, and polymers, are used. These conditions cause different pollutants such as vapors and fumes to emit into the air of workplaces. Some materials are raw materials, some are impurities and some are produced as a secondary product during the production process. Based on the investigations performed by researchers in this factory, some volatile organic compounds such as benzene, toluene, xylene, ethyl benzene, 1,2,3 trimethyl benzene, styrene, 2-propanol, and 2-butoxyethanol, some of the monomers such as vinyl chloride, and some of the fumes such as iron oxide, copper, manganese, zinc oxide, aluminum, and chromium are released into the air in this factory, which can affect the health of workers in various shops.

### 2.2 Sampling and measurement method

Pollutants were sampled in winter and in the time period between 8:00 to 16:00 during the work shift of the workers. Air samples of BTEX were gathered from the breathing zone of participants based on the method of NIOSH 1501 for benzene, toluene, ethyl benzene, and xylene, method of Analytical 369 for 1,2,3 trimethyl benzene and styrene, method of NIOSH 1400 for 2-propanol, method of NIOSH 1403 for 2-butoxyethanol, NIOSH 1007 for vinyl chloride, and NIOSH7303 for metal fumes. Vapors were gathered by the adsorbent tube of activated coconut charcoal and fumes were collected by mixed cellulose esters (MCE) membrane filter produced by SKC company. For this purpose, the adsorbent tube of the cassette filter holder was placed on the worker's collar in the worker’s breathing zone and the air was passed through it using a pump. The range of flow rate for vapors was between 0.05 and 0.20 L per min and that for fumes was between 1 and 4 L per min. Sampling was performed by a personal sampling pump of model AirChek TOUCH (5–5000 mL min^−1^, SKC, Inc.). Firstly, the pretest was conducted to reveal the breakthrough volume. Three samples were collected from the breathing zone of each subject so which covered the whole work shift. At the end of sampling, the open sides of the adsorbent tubes were sealed with plastic caps and transferred into a cool box for preventing sample leakage. Also, the open sides of the cassette filter holder were closed by plastic plugs. After moving the collected samples to the laboratory, the each of adsorbent tubes was broken and the contaminants were chemically desorbed using a related solvent in an extraction vial. To complete extraction, it was allowed that adsorption is performed for 60 min. Then, the extracted sample was injected into a gas chromatograph with flame-ionization detection (GC-FID) (7890 gas chromatograph, and 5975 mass spectrometers, Agilent Technologies, CA, USA) using a syringe. Helium with a flow rate of 1 mL min^−1^ was used as the mobile phase. Each cassette filter holder also was opened and the sample filter was removed with forceps. Then, the filter was transferred to a 50-mL hot block digestion tube and digested by hydrocholeretic acid and nitric acid. After that, prepared samples were analyzed using an atomic absorption spectrometer (SpectrAA 220FS, Varian, CA, USA). In addition to the main samples, a number of blank samples were analyzed for eliminating errors during the steps of sampling, transmitting, and preparing.

### 2.3 Health risk assessment

#### 2.3.1 Non-cancer health risk assessment using the US EPA method

To evaluate the non-cancer risk of the pollutants, the method proposed by the United States environmental protection agency (USEPA) was applied. In this technique, hazard quotient (HQ) is computed as the ratio between exposure to a pollutant and its reference dose for non-carcinogenic effects. The HQ was obtained by equation (1) [3].

(1) HQ = EC/RfC

Where EC is exposure concentration (mg/m^3^) and RfC is the maximum acceptable dose of daily exposure (mg/m^3^). EC also was calculated by equation (2) [3].

(2) EC = (C × ET × ED × EF)⁄AT

where C is the concentration of pollutant (mg/m^3^); other variables are presented in Table 1.

**Table 1:** Variables used in non-carcinogenic and carcinogenic risk assessment.

Variable	Description	Value	United	Reference
C	Concentration of pollutants	–	mg m^−3^	Sampling
IR	Inhalation rate, adult	16	m^3^ day ^−1^	EPA 2011
ET	Exposure time	8–11	Hours day^−1^	Questionnaire
EF	Exposure frequency	275–315	days years^−1^	Questionnaire
ED	Exposure duration	27–32	years	Questionnaire
BW	Body weight	62–96	kg	Questionnaire
RfC	Reference concentration from inhalation	0.03 for benzene 5.00 for toluene 1.00 for ethyl benzene 0.10 for xylene 1.60 for 2-butoxyethanol 0.20 for 2-propanol 0.10 for vinyl chloride 0.06 for 1,2,3 trimethylbenzene 1.00 for styrene 3.063 for iron oxide 0.162 for copper 0.00005 for manganese 1.312 for zinc oxide 0.005 for aluminum 0.0001 for chromium	mg m^−3^	IRIS IRIS IRIS IRIS IRIS IRIS IRIS IRIS IRIS IRIS IRIS IRIS IRIS IRIS IRIS
AT	Average lifetime	9000	days	[22]
SF	slop factor	0.029 for benzene 0.0087 for ethyl benzene 0.30 for vinyl chloride 47.60 for chromium	(mg/kg-day)^−1^	IRIS IRIS RAIS [23]

HQ equal to and less than 1 shows the absence of non-carcinogenic health effects and HQ more than 1 revealed the presence of non-carcinogenic health effects [24].

#### 2.3.2 Cancer health risk assessment using the US EPA method

To examine the cancer risk of the pollutants, the method suggested by the US environmental protection agency (USEPA) and its database (the integrated risk information system (IRIS)) was exploited. In this method, the lifetime cancer risk (LCR) index was calculated to estimate the carcinogenic risk of exposure to benzene and ethylbenzene vapors. The value of the index was estimated using Equation (3) [3].

(3) LCR = CDI × SF

where SF is slop factor (kg.day.mg ^−1^) and CDI is chronic daily intake (mg.kg^−1^. day). SF was extracted from the database of the IRIS [25,26]. CDI was also computed using equation (4) [3].

(4) $$CDI=\frac{C\times IR\times ED\times EF}{BW\times AT}$$

where C is the concentration of pollutant (mg m^−3^); other variables are presented in Table 1.

### 2.4 Statistical analysis

In this study, SPSS was applied for statistical analysis.

## 3 Results

Table 2, Table 3 show the concentration of vapors and fumes in the breathing zone of the workers participating in this study, respectively. Also, the threshold limit values (TLVs) of these pollutants according to the American conference of governmental industrial hygienists (ACGIH) have been reported in these tables [24]. Based on the results, the concentration of copper in the press shop, the concentration of manganese in the body shop, the concentration of 2-butoxyethanol in the paint shop, toluene, and manganese in the pre-delivery inspection shop, and benzene in the assembly shop were found to be higher than the TLVs. In total, the concentration of 2-butoxyethanol, copper, and manganese was greater than TLVs. Given that the TLVs have been recommended for 8 h of work per day and 5 days of work per week, the amount of TLV-TWA was corrected using the Scala brief model if the work duration was more than 40 h per week [27].

**Table 2:** The concentration of vapors in the breathing zone of the workers.

**Table 3:** The concentration of fumes in the breathing zone of the workers.

Table 4 describes the values of HQ in non-cancer risk assessment of pollutants as a heat map. The results revealed that the values of non-cancer risk (HQ) due to exposure to vapors of benzene in shops of press, paint, predelivery inspection, assembly, and material storage, toluene in the shop of predelivery inspection, ethyl benzene in the shop of predelivery inspection xylene in shops of body, pre-delivery inspection, and assembly, 2-butoxyethanol in the shop of paint, 1,2,3-trimethylbenzene in the shop of paint were higher than the acceptable limit (1). Among fumes, the results showed that the values of non-cancer risk (HQ) due to exposure to copper in the shop of the press, manganese in shops of body and pre-delivery inspection, aluminum in shops of body and pre-delivery inspection, and chromium in the shop of the body were greater than the acceptable limit (1). Based on the results, the concentrations of the values of non-cancer risk (HQ) due to exposure to vinyl chloride, styrene, iron oxide, and zinc oxide were not higher than the acceptable limit in any of the shops.

**Table 4:** The values of HQ in non-cancer risk assessment of pollutants as a heat map.

Table 5 represents the statistical values of LCR for benzene, ethylbenzene, vinyl chloride, and chromium as a heat map. It is necessary to state that only data for benzene, ethylbenzene, vinyl chloride, and chromium are given as the SF for other substances in this study is not available. The results showed that the estimated LCR for benzene was higher than 10^−5^ in shops of press, body, paint, and material storage and it was greater than 10^−4^ in shops of predelivery inspection and assembly. The calculated LCR for ethyl benzene also was higher than 10^−5^ in shops of press, body, and paint and greater than 10^−4^ in shops of predelivery inspection and assembly. Based on the results, the values of computed LCR related to vinyl chloride and chromium were higher than 10^−4^ in the paint shop and greater than 10^−2^ in the body shop, respectively. The results indicated that the values of LCR of these substances are higher than the threshold risk level (10^−6^) set by the US EPA in stated shops. These results clearly show the potential carcinogenicity risk to workers in the shops of this industry.

**Table 5:** Cancer risk assessment of vapors and fumes in the breathing zone of the workers.

## 4 Discussion

This study aimed to evaluate the health risk of exposure to various vapors and fumes in a factory of an automobile manufacturer. The results showed the concentration of copper in the press shop, the concentration of manganese in the body shop, the concentration of 2-butoxyethanol in the paint shop, toluene, and manganese in the pre-delivery inspection shop, and benzene in the assembly shop were found to be higher than the TLVs. In the body shop, welding is performed which can lead to the release of various metal fumes into the air [28]. Based on the type of base metal, the type of welding, the type of fumes, and their concentration are different [29]. In this study, the concentration of manganese fume was higher than TLV among different fumes. The results of a study performed by Mansouri et al. showed that the concentration of manganese fume in the breathing zone of welders using coated electrodes in an automobile part manufacturing factory in Iran was 0.18 mg/m^3^, which is close to TLV while the concentration of chromium and zinc were very low for detecting [30]. The results of a study conducted by Li in a vehicle factory in China also indicated that the concentration of manganese was 1.45 mg/m^3^, which is higher than TLV [31]. Mehrifar et al. concluded that exposure to manganese fume is associated with neurological, neuropsychological, and pulmonary adverse health effects [32]. Based on the results of the present study, the concentration of 2-butoxyethanol was higher than TLV in the paint shop. This substance is widely used as a solvent in cleaning and painting [33]. Lee et al. also concluded that four chemicals, including toluene, ethylbenzene, xylene, and 2-butoxy ethanol may pose health risks in workers that use car colorant products [34]. 2-butoxy ethanol is known as a respiratory irritant and a suspected human carcinogen [35]. In this report, concentrations of toluene and manganese in the pre-delivery inspection shop were greater than TLV. In this part, the car body is inspected and defects are fixed. For this purpose, the steps of welding, painting, and varnishing may be performed. Exposure to manganese fume can occur in the step of welding and exposure to BTEX can happen during the use of paints and solvents. In the study of Martins et al., toluene presented higher indoor concentrations and indoor and outdoor ratios, showing that the paint and varnish workplaces had significant BTEX sources [36]. The central nervous system is affected and weakened by toluene. Drowsiness, fatigue, dizziness, and nausea are toxic effects of toluene. Tremors, mental and nervous disorders, movement imbalance, and ataxia have also been described as a result of exposure to this substance. In addition, atrophy of nerve cells, vision and hearing disorders, hallucinations, and depression have been reported [37]. Based on the results of the present study, the concentration of benzene in the assembly shop was higher than TLV. Benzene may be in solvents and adhesives in this part. A wide variety of industries and occupations use benzene or benzene-containing solvents and adhesives which can affect the health of workers [38]. Also, Lexen et al. concluded that total volatile organic compounds (VOCs) concentrations were initially very high in cars which reached the market one to two months after manufacture [39]. In a study performed by Harati et al., the exposure to benzene was higher than TLV (0.5 PPM) among workers in an automobile manufacturing factory in Iran [40]. benzene affects the central nervous can lead to euphoria, headache, nausea, movement imbalance (ataxia), convulsions, and coma. Arrhythmia also occurs due to the sensitivity of the heart to catecholamines. Some of the signs and symptoms of chronic poisoning with benzene include weakening bone marrow, leukopenia, and thrombocytopenia [41].

It should be noted that although the concentration of some substances was lower than TLV, some of them were associated with non-carcinogenic health risks. The results of the present study showed that the values of non-cancer risk (HQ) due to exposure to vapors of benzene in shops of press, paint, predelivery inspection, assembly, and material storage, toluene in the shop of predelivery inspection, ethyl benzene in the shop of predelivery inspection, xylene in shops of body, pre-delivery inspection, and assembly, 2-butoxyethanol in the shop of paint, 1,2,3-trimethylbenzene in the shop of paint were higher than the acceptable limit. These VOCs are present in solvents, paints, and adhesives in various shops in the car manufacturing industries. Because different processes, such as cleaning, welding coated metals, painting, and assembling are performed in this type of industry which can lead to the production or vaporization of these substances [3]. The results of a study performed by Tong et al. showed that the total non-carcinogenic risk in automobile manufacturing processes was the highest in the paint shop [3]. Moreover, Wang et al. identified the important VOCs emitted from different processes in a typical automobile manufacturing enterprise, including benzene, toluene, xylene, ethyl benzene, 2-butoxyethanol, 1,2,4 trimethyl benzene, isopropanol, heptane, and butanone [42]. Dehghani et al. also evaluated the health risk of BTEX in the paint unit of an automobile manufacturing industry. They concluded that the non-cancer risk for benzene in the paint unit was higher than recommended allowable level while the non-cancer risk for toluene, xylene, and ethyl benzene was lower than recommended allowable level [43]. Loonsamrong et al. also performed a study on health risk assessment of BTEX exposure among car park workers. The results of this study showed that the non-cancer risks for benzene, toluene, ethylbenzene, and xylene were within acceptable limits [44]. Harati et al. also conducted a study on risk assessment of exposure to chemical pollutants in the automobile manufacturing industry. They concluded that there is a high risk of non-cancer effects from exposure to benzene [45]. Farshad et al. also evaluated the risk of exposure to BTEX in paint plants of two automotive industries. Based on the results of this study, the maximum risk was for benzene and toluene, respectively and those were higher than acceptable values [46]. The results of the present study were nearly similar to the findings of previous studies. However, these studies have often been performed in the paint unit of automobile manufacturing industries. Among fumes, the results of the present study showed that the values of non-cancer risk (HQ) due to exposure to copper in the shop of the press, manganese in shops of body and pre-delivery inspection, aluminum in shops of body and pre-delivery inspection, and chromium in the shop of the body were greater than the acceptable limit. However, the results of the present study indicated that the concentrations of the values of non-cancer risk (HQ) due to exposure to vinyl chloride, styrene, iron oxide, and zinc oxide were not higher than the acceptable limit in any of the shops. Bakri et al. also investigated the toxicity of metal fumes and their relationship with lung health problems among welders in the automotive industry. They concluded that there is strong evidence of the progression of lung disease among welders in this industry because of exposure to metal fumes of nickel, chromium, iron, aluminum, cadmium, and beryllium [29]. Nduka et al. estimated the health risk of cadmium, chromium, and nickel in car paint used at auto-panel workshops. They concluded that the highest hazard quotients were related to cadmium [20]. In another study, Nduka et al. assessed the human health risk of exposure to lead, manganese, and copper from scrapped car paint dust from automobile workshops. Based on the results of this study, the highest hazard quotients were assigned to lead and manganese [47]. The results of these studies are different from the findings of the present study. It can be because there are various fumes in different workplaces. However, in total, the results of the previous studies and the present study showed that exposure to fumes can be associated with serious non-cancer effects.

The results of the present study showed that the estimated LCR for benzene in shops of press, body, paint, material storage, pre-delivery inspection, and assembly, for ethyl benzene in shops of press, body, paint, pre-delivery inspection, and assembly were higher than the threshold risk level set by the US EPA. In the other words, the results indicate the potential carcinogenicity risk of these substances in the shops of the automobile manufacturing industry. The results of a study performed by Badjagbo et al. revealed that benzene levels are a potential cancer risk for the workers occupied as automobile mechanics and painters [21]. Tong et al. also concluded that there are large carcinogenic risks in various processes of automobile manufacturing factories because of exposure to the high concentrations of benzene and ethylbenzene among VOCs, suggesting a definite risk [3]. Dehghani et al. also assessed the health risk of BTEX in the paint unit of an automobile manufacturing industry. In this study, the values of cancer risk for benzene and ethyl benzene per 1000 have been reported by 1.27 and 0.39, respectively [43]. Loonsamrong et al. also conducted a study on health risk assessment of BTEX exposure among car park workers. Based on the results of this study, the cancer risk for exposure to benzene and ethyl benzene was over acceptable levels [44]. Harati et al. also conducted a study on risk assessment of exposure to chemical pollutants in the automobile manufacturing industry. The results of this study showed that there is a risk of leukemia in workers exposed to benzene [48]. The results of the present study were nearly similar to the findings of previous studies. However, these studies have often been performed in the paint unit of automobile manufacturing industries. Moreover, the results of the present study indicated that the estimated LCR for vinyl chloride in the paint shop and chromium in the body shop was higher than the threshold risk level set by the US EPA. Kahforoushan et al. estimated the risk assessment of exposure to pollutants in automobile tire manufacturing factories. In this study, vinyl chloride was introduced as the most dangerous chemical material, which has a high risk of carcinogenicity [49]. The results of a study conducted by Bakri et al. revealed that the cancer risk of inhalation exposure to chromium fume in welding was higher than the threshold risk level set by the US EPA [50]. Nduka et al. also performed a study on the health risk assessment of cadmium, chromium, and nickel in car paint used at auto-panel workshops. Based on the results of this study, inhalation exposures to these substances can add to the body's burden of some carcinogenic [20]. These results are consistent with the findings of the present study.

As a limitation, there is uncertainty in the health risk assessment in data and analyses because the nature of risk assessment is probabilities. However, there are uncertainties in all scientific studies. It is suggested that the Monte-Carlo simulation is used in the next studies to decrease this uncertainty. Therefore, the results of such studies can be used as a guide for hazard perception and those must not be considered as criteria for estimating the definite health effects.

## 5 Conclusion

The concentration of copper, manganese, 2-butoxyethanol, toluene, and benzene in some shops was found to be higher than the TLVs. Although the concentration of some substances was lower than TLV, the values of non-cancer risk (HQ) due to exposure to vapors of benzene, toluene, ethyl benzene, xylene, 2-butoxyethanol, 2-propanol, 1,2,3-trimethylbenzene, copper, manganese, aluminum, and chromium in some shops were higher than the acceptable limit. Moreover, the results showed that the estimated LCR for benzene, ethyl benzene, vinyl chloride, and chromium in some shops were higher than the threshold risk level set by the US EPA. The results demonstrated that the occurrence of cancer and non-cancer consequences because of exposure to the stated substances is serious. It is emphasized that control measures, such as preparation of general and local ventilation, the enclosure of the production process to decrease the air born concentrations, in addition to the use of personal protective equipment.

## Declarations

### Ethics approval and consent to participate

The protocol was reviewed and approved by the Medical Ethics Committee of Kashan University of Medical Sciences (IR.KAUMS.NUHEPM.REC.1400.048). All steps of the study were in accordance with the ethical standards. All participants were asked to fill out the consent form developed by the medical ethics committee, and written informed consent was obtained from all of them.

## Author contribution statement

Amir Hossein Khoshakhlagh: Performed the experiments; Wrote the paper.

Saeid Yazdanirad: Conceived and designed the experiments; Analyzed and interpreted the data; Wrote the paper.

Hamid Reza Saberi: Performed the experiments; Contributed reagents, materials, analysis tools or data.

Pao-Chi Liao: Analyzed and interpreted the data.

## Data availability statement

The authors do not have permission to share data.

## Declaration of competing interest

The authors declare that they have no known competing financial interests or personal relationships that could have appeared to influence the work reported in this paper
